# Need of Treatment Modification During Entecavir Therapy in Patients with Chronic Hepatitis B: Long-Term Follow-Up Results for 120 Months

**DOI:** 10.3390/microorganisms13020218

**Published:** 2025-01-21

**Authors:** Hae Rim Kim, Seong Hee Kang, Hyung Joon Yim, Sang Jun Suh, Young Kul Jung, Ji Hoon Kim, Yeon Seok Seo, Jong Eun Yeon, Kwan Soo Byun

**Affiliations:** Department of Internal Medicine, Korea University College of Medicine, Seoul 02841, Republic of Korea; haerami@gmail.com (H.R.K.); shkang0114@gmail.com (S.H.K.); mothpickle@naver.com (S.J.S.); 93cool@hanmail.net (Y.K.J.); kjhhepar@naver.com (J.H.K.); drseo@korea.ac.kr (Y.S.S.); jeyyeon@hotmail.com (J.E.Y.); byunks@gmail.com (K.S.B.)

**Keywords:** entecavir, hepatitis B, high viral load, partial virologic response

## Abstract

Entecavir (ETV) may have limited antiviral efficacy in chronic hepatitis B (CHB) patients with a high baseline viral load, especially in cases of partial virologic response (PVR). This study evaluated the outcomes of prolonged ETV monotherapy, given the lack of clear evidence favoring continuation or combination therapy in such scenarios. We included 188 treatment-naïve patients on ETV 0.5 mg and compared antiviral responses between high viral load (HVL, ≥7 log10 IU/mL) and non-HVL groups for over up to 120 months in this study. Compared to the non-HVL group, the HVL group exhibited a lower cumulative virologic response (VR, <20 IU/mL) during the 10-year therapy period (*p* < 0.01). Antiviral resistance to entecavir (rtS202G + rtM204V + rtL180M) developed in three out of 90 patients in the HVL group. Patients with complete response at 6 months had a 100% likelihood of achieving VR, while 83.9% of patients with PVR at 6 months achieved VR by month 120 (*p* < 0.01). Multivariate analysis revealed that baseline HVL was a significant predictor of long-term VR at 120 months. In conclusion, CHB patients with baseline HVL and PVR at 6 months are prone to ETV resistance and inadequate response, warranting a modification in treatment strategy.

## 1. Introduction

Long-term complications of chronic hepatitis B (CHB) encompass liver cirrhosis and hepatocellular carcinoma (HCC), which are associated with persistence of viral replication [1]. Achieving sustained viral suppression to an undetectable hepatitis B virus DNA level is imperative for preventing such complications, necessitating prolonged antiviral therapy with nucleos(t)ide analogs in many CHB patients [2,3,4].

Entecavir (ETV) is recommended as the initial antiviral agent for treatment-naïve CHB patients due to its potent antiviral activity and high genetic barriers [5]. While the rate of virologic response (VR), defined as undetectable HBV DNA by PCR, is significantly higher with ETV compared to first-generation nucleos(t)ide analogs (NAs), not all patients achieve VR during long-term ETV therapy [6,7]. Considering that antiviral response tends to be unfavorable in patients with a baseline high HBV DNA level receiving NAs, further evaluation of responses to ETV in this group is warranted to assess the need for treatment strategy modification [8,9,10].

Contrarily, findings from a study involving HBV patients treated with tenofovir disoproxil fumarate (TDF) for 5 years indicate that 99.2% of patients with a baseline HBV DNA < 9 log_10_ copies/mL achieved HBV DNA < 400 copies/mL, while 98.3% of those with a baseline HBV DNA > 9 log_10_ copies/mL achieved the same level of suppression [11]. This suggests that TDF monotherapy could result in virological suppression in most CHB patients, even those with high viral loads (HVL), although achieving virological suppression might take longer in patients with HVL. Nevertheless, ETV remains a crucial first-line treatment option due to its excellent safety profile, particularly in patients with impaired renal function, bone diseases, dyslipidemia, or elderly patients.

On-treatment response is also pivotal in predicting long-term VR after anti-HBV therapy [8,12,13]. Detecting HBV DNA after 12 months of ETV treatment, deemed a partial virological response (PVR), holds prognostic significance [7]. If PVR occurs despite patient compliance with a given medication, alternative strategies may need to be considered.

This study aimed to assess the antiviral efficacy of ETV therapy in treatment-naïve patients with CHB undergoing 10 years of continuous treatment, with a focus on outcomes in difficult-to-treat populations with baseline HVL and/or PVR to determine the appropriateness of continuing monotherapy.

## 2. Materials and Methods

### 2.1. Study Population

This retrospective study included consecutive treatment-naïve patients with chronic hepatitis B (CHB) who were aged 18 years or older and had tested positive for hepatitis B surface antigen (HBsAg) for at least 6 months. These patients initiated treatment with entecavir (ETV) 0.5 mg between January 2007 and March 2012. The inclusion criteria, based on established guidelines for antiviral therapy in CHB, were as follows: (a) HBeAg-positive CHB: ALT >2× the upper limit of normal (ULN) or moderate to severe hepatitis on biopsy, with HBV DNA >20,000 IU/mL; (b) HBeAg-negative CHB: HBV DNA >20,000 IU/mL and ALT >2× ULN; (c) compensated cirrhosis: ALT >2× ULN or high HBV DNA levels (>2000 IU/mL), regardless of ALT levels; (d) decompensated cirrhosis: detectable HBV DNA, regardless of ALT levels.

Exclusion criteria were (a) diagnosed hepatocellular carcinoma at the enrolment; (b) transplant surgeries other than liver or kidney; (c) discontinuing medication before six months of treatment; (d) presence of serum antibodies against hepatitis C virus, hepatitis D virus, or human immunodeficiency virus. Among the 318 patients treated with ETV during the study period, 130 were excluded based on the aforementioned criteria. Ultimately, data from 188 patients were analyzed in this study (Supplementary Figure S1). Patients with a creatinine clearance of more than 50 mL/min were administered with 0.5 mg of ETV daily, regardless of body weight. Antiviral treatment was continued indefinitely unless adverse effects required discontinuation, with no supplementary treatment.

Patients’ medical records were reviewed. Approval for this study was obtained from the Institutional Review Board of Korea University Ansan Hospital [AS13184-001], conducted in accordance with the ethics code of the World Medical Association (Declaration of Helsinki).

#### 2.1.1. Laboratory Testing

Patients underwent routine laboratory tests, including serum creatinine and HBV-DNA levels as well as other viral markers that were monitored at 3–6-month intervals. Patients were screened for HCC and cirrhotic complications every 6 months using ultrasonography and serum alpha-fetoprotein levels. Serum HBV DNA testing employed the TaqMan real-time PCR assay (COBAS TaqMan, Roche Molecular System, Branchburg, NJ, USA) with a lower detection limit of 20 IU/mL. Genotypic resistance to ETV was determined using restriction fragment mass polymorphisms (RFMP; Green Cross Reference Laboratories, Yongin, Korea) as previously described [14]. Briefly, the RFMP method involves amplifying the drug resistance mutation regions via PCR, followed by restriction enzyme digestion to fragment the amplified DNA into oligonucleotides.

#### 2.1.2. Definitions

VR was defined as an undetectable HBV DNA level (<20 IU/mL) during treatment. Early VR is defined based on the VR at the 6-month time point regardless of HBeAg and biochemical response status, whereas PVR is defined as a decrease in HBV DNA of more than 1 log_10_ IU/mL but with detectable HBV DNA after at least 6 months of therapy. Virological breakthrough was defined as an increase in serum HBV DNA level > 1 log_10_ IU/mL from the nadir. Biochemical response (BR) indicated a decrease in ALT levels below the upper normal limit (45 U/L) in both sexes. Serological responses comprised loss of HBeAg and seroconversion of HBeAg to anti-HBe. Baseline high viral load (HVL) was defined as >7 log_10_ IU/mL at antiviral therapy initiation. PVR6 and PVR12 signified HBV DNA detection using real-time PCR after 6 and 12 months of ETV treatment, respectively.

HBV mutation test was performed in cases where there was initial viral suppression following ETV treatment, but a virologic breakthrough subsequently occurred. ETV mutation was defined as follows: the presence of M204V  +  L180M mutations, along with one of the following additional mutations: T184A/G/I/L/S, S202G, or M250V.

Cirrhosis was diagnosed on the basis of clinical findings: (a) platelet count of less than 100,000/µL and ultrasonography findings suggestive of cirrhosis, including a blunted, nodular liver edge accompanied by splenomegaly (bipolar diameter >12 cm) or (b) clinical signs of portal hypertension, such as ascites, esophageal or gastric varices, and hepatic encephalopathy.

Clinical and laboratory data were compared between patients with baseline HVL (HVL group) and those without HVL (non-HVL group). The primary endpoint of this study was cumulative VR during 120 months of therapy. Secondary endpoints included biochemical response, serological response, and antiviral resistance during the same period. The index date was the date of the first prescription of ETV and the follow-up period was between the index date and the date of switch to a different medication, death, liver transplantation, or the final follow-up.

#### 2.1.3. Statistical Analysis

Data are presented as mean ± standard deviation or n (%). Student’s *t*-test and paired *t*-test were used for continuous variables, while a chi-square test was used for categorical variables. The Mann–Whitney U test was employed for subgroup analysis with a small population. A Kaplan–Meier survival analysis and a log-rank test compared cumulative VR rates. Covariates with *p* < 0.05 in univariate analysis were included in multivariate analysis using the Cox proportional hazards model to predict VR during treatment. Variables included sex, age, presence of HVL, ALT, total bilirubin, HBeAg positivity, albumin level, PT (INR), and VR at 12 and 24 months. Given the interaction between HVL and VR during treatment and multicollinearity among VR at different time points, three models were used. Statistical significance was set at *p* < 0.05.

## 3. Results

### 3.1. Baseline Characteristics

A total of 188 patients were included in this analysis, and their baseline characteristics are presented in Table 1. The mean age of the patients was 44.03 ± 10.97 years. The mean serum HBV DNA at baseline was 7.72 ± 0.45 and 5.58 ± 1.03 log_10_ IU/mL in the HVL and non-HVL groups, respectively. The proportion of patients with HBeAg was higher in the HVL group than in the non-HVL group (72/90, 80.0% vs. 34/98, 34.7%, *p* < 0.01). Conversely, the proportion of patients with cirrhosis was lower in the HVL group compared to the non-HVL group (16/90, 17.8% vs. 57/98, 58.2%, *p* < 0.01).

### 3.2. Antiviral Response

**Figure 1:**
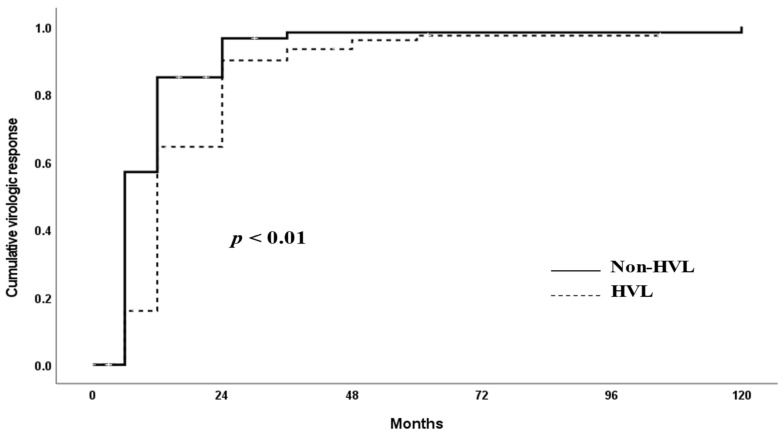
Cumulative virological response in HVL and non-HVL groups.

Most patients followed up for 120 months of treatment exhibited long-term virological response (VR, 90.8%). At months 12, 24, 48, 60, and 120 of ETV treatment, VR was achieved in 43.2% (38/88), 67.9% (55/81), 79.7% (51/64), 84.7% (50/59), and 84.0% (42/50) of patients in the HVL group and 87.2% (82/94), 93.3% (83/89), 96.1% (73/76), 97.1% (67/69), and 96.6% (57/59) of patients in the non-HVL group, respectively. The HVL group demonstrated lower VR rates than the non-HVL group at all time points (*p* < 0.05) (Table 2). The cumulative VR up to 120 months was higher in the non-HVL group than in the HVL group (*p* = 0.02) (Figure 1). The HVL group exhibited a significantly higher partial virological response (PVR) rate at 6 months (41.8% vs. 83.3%, *p* < 0.01) and at 12 months (12.8% vs. 56.8%, *p* < 0.01) compared to the non-HVL group.

### 3.3. Serologic Response

At 120 months, HBeAg loss rates were 100% (20/20) and 78.9% (30/38) in the non-HVL group and the HVL group, respectively (*p* = 0.03) (Supplementary Figure S2A). In the HVL group, the rate of HBeAg loss was lower than that in the non-HVL group from the 36-month time point after ETV treatment (*p* < 0.05). HBeAg loss rates in both groups continued to increase throughout the study period. HBeAg seroconversion rates at 120 months were 40.0% (8/20) and 36.8% (14/38) in the HVL and non-HVL groups, respectively, with no significant difference between the groups (*p* = 0.81) (Supplementary Figure S2B).

### 3.4. Biochemical Response

Most patients followed up for 120 months of treatment demonstrated BR (91.3%). At months 12, 24, 48, 60, and 120 of ETV treatment, BR was achieved in 81.8% (72/88), 90.2% (74/82), 93.7% (59/63), 84.5% (49/58), and 93.3% (40/42) of patients in the HVL group and 86.2% (81/94), 90.0% (81/90), 94.7% (72/76), 85.9% (61/71), and 88.7% (55/62) of patients in the non-HVL group, respectively. There were no differences in the BR rates between the HVL and non-HVL groups (Supplementary Table S1).

### 3.5. Resistance to Entecavir

During the 120-month study period, antiviral resistance to ETV (rt202G + rt204V + 180M) was observed in three patients (3.3%) in the HVL group and none (0%) in the non-HVL group. Although not statistically significant (*p* = 0.07), the HVL group tended to have higher resistance rates than the non-HVL group. Among these patients, all experienced both PVR6 and PVR12. One patient exhibited antiviral resistance after 60 months of therapy, while the other two developed resistance within 120 months. Upon confirmation of resistance, one patient transitioned to TDF, while the other two switched to tenofovir alafenamide (TAF), with all three achieving virologic response 3 to 12 months after the change in therapy.

### 3.6. PVR and Long-Term VR at 120 Months

**Figure 2:**
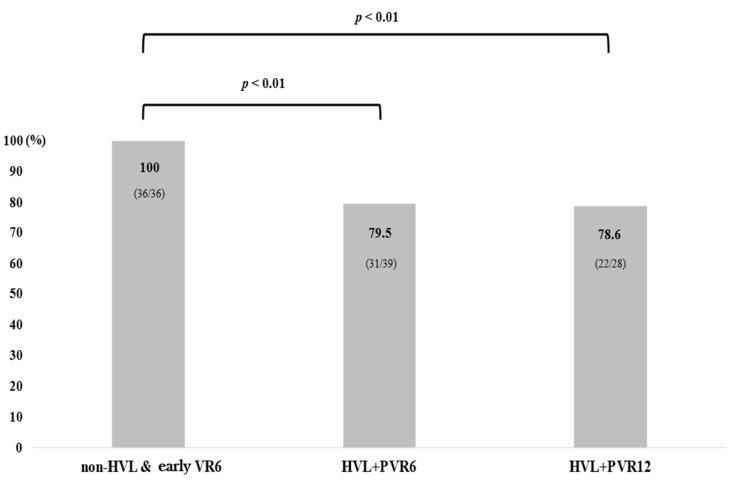
Virological response at 120 months according to baseline viral load or PVR at 6 months and 12 months. Group 1: patients with baseline non-HVL and early VR6; Group 2: patients with both baseline HVL and PVR at 6 months; Group 3: patients with both baseline HVL and PVR at 12 months.

We conducted an analysis of VR at 120 months based on baseline HBV DNA levels and PVR6 or PVR12. The VR rate at 120 months was 100% (36/36) in patients without HVL or PVR (Group 1), 79.5% (31/39) in those with both baseline HVL and PVR at 6 months (Group 2), and 78.6% (22/28) in patients with both baseline HVL and PVR at 12 months (Group 3) (Figure 2). In the non-HVL group, when comparing VR rates at 120 months according to PVR6, the VR rate was 100% (36/36) in patients without PVR6, but it was lower at 91.3% (21/23) in those with PVR at 6 months, although this difference was not statistically significant (Supplementary Figure S3). Further analysis aimed to determine whether the presence of PVR6 predicted failure to achieve VR at 120 months (Table 3). Throughout the treatment duration, VR was achieved in 45.9% (51/111), 71.6% (73/102), 83.1% (69/83), 84.9% (62/73), and 83.9% (52/62) of patients in the PVR6 group at months 12, 24, 48, 60, and 120, respectively, compared to 97.2% (69/71), 95.6% (65/68), 96.5% (55/57), 100% (55/55), and 100% (47/47) of patients with a complete response at 6 months group. Patients with a complete response at 6 months had a significantly higher long-term VR rate than those with PVR at 6 months at all time points (all *p* < 0.01). Consequently, patients with a complete response at 6 months had a 100% chance of achieving long-term VR at 120 months with continued treatment, while patients with PVR at 6 months had an 83.9% chance of achieving long-term VR at 120 months.

### 3.7. Factors Predictive of Long-Term VR

Univariate analysis revealed that long-term VR at 120 months was associated with the presence of HVL, albumin, and prothrombin time (INR) among the variables related to the status at treatment initiation (Table 4). In the multivariate analysis, model 1, adjusted for variables related to the status at treatment initiation, demonstrated that only the presence of HVL (OR, 0.15; *p* = 0.02) was a significant factor. In model 2, adjusted for other variables excluding the presence of HVL at treatment initiation, VR at 12 months (OR, 5.65; *p* = 0.01) remained significant. However, significant Cox analysis results regarding the association between PVR6 and VR at 120 months could not be established, as 100% of patients with a complete response at 6 months achieved long-term VR, compared to 83% in the PVR6 group.

## 4. Discussion

Among the nucleos(t)ide analogs, ETV, TDF, and TAF are recommended as first-line agents [5]. ETV therapy for over 5 years has demonstrated high antiviral efficacy and a substantial rate of histological improvement [6,15].

Our findings indicate that the cumulative VR over 120 months of long-term follow-up was lower in the HVL group compared to the non-HVL group. Previous research on ETV suggested that a baseline HVL (HBV DNA > 8 log_10_ copies/mL) is a negative predictor of VR (HBV DNA < 12 IU/mL) during on-treatment follow-up after three years of treatment; while 100% of patients without HVL achieved undetectable HBV DNA, only 75% of patients with HVL showed similar trend [16]. Another study demonstrated that VR (HBV DNA < 300 copies/mL) was attained in 68% of HVL (HBV DNA > 9 log_10_ copies/mL) patients and 85.71% of non-HVL patients at the end of year two [17]. Despite potential variations in the definitions of HVL and VR, these findings underscore the importance of baseline HVL status in ETV therapy, corroborating our study results.

Primary non-response to ETV, TDF, or TAF is uncommon. Patients who do not respond to these agents after 12–24 weeks should undergo evaluation for compliance, and resistance analyses should be conducted after 24 weeks to identify the presence of drug-resistant variants. Thus, serum HBV DNA levels should be monitored at 24 weeks to confirm ongoing virologic suppression by antiviral therapy. The guidelines from the European Association for the Study of the Liver (EASL) and the American Association for the Study of Liver Diseases (AASLD) address patients with partial virological response (PVR) and suggest that those with declining serum HBV DNA levels may continue treatment with the same agent, given the increasing rates of VR over time and the very low risk of resistance to long-term monotherapy with either ETV or TDF [18,19]. Nonetheless, in this study, patients achieving early VR at 6 months had a 100% likelihood of attaining long-term VR at 120 months with continued treatment, while those experiencing PVR at 6 months still had a 16.1% chance of not achieving VR despite continued therapy. Furthermore, VR at 12 months emerged as a significant predictor of VR at 120 months. These findings underscore the importance of assessing PVR or VR at 6 or 12 months rather than waiting until 24 months for predicting long-term VR.

**Figure 3:**
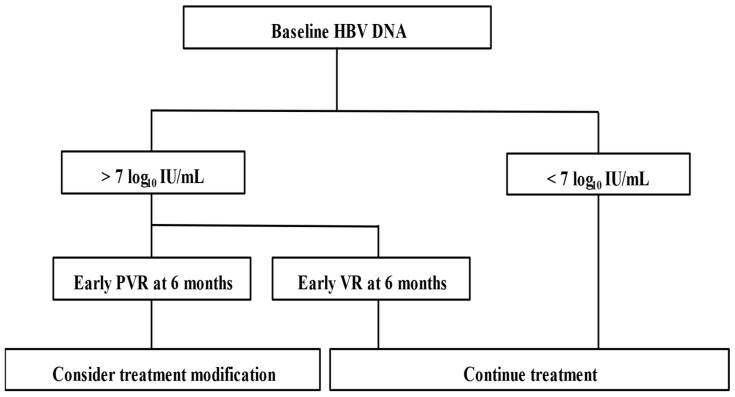
Treatment algorithm for entecavir in CHB patients. Abbreviations: VR, virological response; PVR, partial virological response.

In the multivariate analysis, the predictors of response included pre-treatment characteristics such as non-HVL (HBV DNA < 7 log_10_ IU/mL), albumin levels, and PT INR, while VR at 12 months during treatment was also identified. Previous studies have indicated predictors of response, including viral DNA levels of 2 × 10^8^ IU/mL or lower, elevated serum ALT levels, high activity scores on liver biopsy at baseline, and early viral load reduction within 6 months of treatment [18]. Since all patients in the early VR6 group reached VR, significant Cox analysis results regarding the association between PVR at 6 months and VR at 120 months were not demonstrated in this study. However, PVR at 6 months, along with PVR at 12 months, is anticipated to be an important predictor of response at 120 months. Thus, it is speculated that for patients with HVL treated with ETV and PVR at six months, treatment strategies may need adjustment or switching to more potent antiviral drugs, such as TDF or TAF (Figure 3).

According to a previous report, ETV monotherapy in treatment-naïve patients resulted in a resistance rate that remained at 1.2% after up to 5 years of treatment [15]. Resistance to ETV appeared to occur through a two-hit mechanism, with an initial expression of the M204V/I mutation followed by an amino acid substitution at rtT184, rtS202, or rtM250 [20]. In our study, antiviral resistance to ETV (rt202G, LMV rt204V, 180M) was developed in only three patients in the HVL group (1.5% overall, 4.3% in HVL group). The AASLD guidelines indicate that the rate at which resistant variants are selected is related to the pretreatment serum HBV DNA level, rapidity of viral suppression, and duration of treatment [19]. This underscores the importance of baseline viral load and early virologic response in preventing the occurrence of resistance.

HBeAg seroconversion represents a crucial treatment goal for HBeAg-positive patients with CHB, indicating a favorable prognosis characterized by reduced rates of cirrhosis and slower disease progression [5,21]. In our study, we observed HBeAg/anti-HBe seroconversion rates of 26.3% and 40% in the HVL and non-HVL groups, respectively, at 12 months. Previous research suggested that HBeAg seroconversion rates with ETV ranged from 15% to 21% after one year of treatment and from 21% to 31% after two years of treatment [22,23,24]. Even in a long-term follow-up study spanning over 10 years, it was shown that low baseline HBV DNA levels in patients receiving ETV are pivotal predictors of HBeAg seroconversion.

Based on these findings, we recommend adhering to the HBV treatment algorithm, which tailors treatment strategies according to early predictive indicators of efficacy at six months. Continuation of therapy with the same drug is advisable for patients exhibiting VR, while those with baseline HVL and PVR at six months consider treatment modification. This may involve switching to TDF, TAF monotherapy, TDF, TAF plus ETV combination therapy, or close monitoring [25].

The limitations of our study include the small number of patients, retrospective design, and missing data during the 120-month follow-up period. Further prospective, large-scale cohort studies are warranted.

In conclusion, although continued treatment with ETV proved highly efficacious in achieving VR, baseline HVL was associated with a lower response rate in the 120-month long-term follow-up results. Observation of PVR at 6 months increased the risk of failing to achieve VR and exposed patients to antiviral resistance. We suggest that CHB patients with baseline HVL and PVR consider modifying their treatment strategies during ETV therapy.

## Figures and Tables

**Table 1:** Baseline characteristics of patients in the non-HVL and HVL groups.

	All (*n* = 188)	Non-HVL (<10^7^ IU/mL) (*n* = 98)	HVL (≥10^7^ IU/mL) (*n* = 90)	*p*
Male, n (%)	124 (66.0%)	67 (68.4%)	57 (63.3%)	0.46
Age (years)	44.03 ± 10.97	46.55 ± 9.85	41.28 ± 11.51	<0.01
HBV DNA (log_10_ IU/mL)	6.60 ± 1.34	5.58 ± 1.03	7.72 ± 0.45	
HBeAg-positive	106 (56.4%)	34 (34.7%)	72 (80.0%)	<0.01
ALT (IU/L)	195.54 ± 303.07	161.83 ± 286.11	232.24 ± 318.05	0.11
Total bilirubin (mg/dL)	1.59 ± 2.06	1.62 ± 1.99	1.57 ± 2.15	0.87
Albumin (g/dL)	3.97 ± 0.56	3.97 ± 0.55	3.97 ± 0.58	0.97
PT, INR	1.10 ± 0.20	1.13 ± 0.24	1.08 ± 0.14	0.08
Cr	1.04 ± 0.26	1.05 ± 0.29	1.03 ± 0.22	0.70
Cirrhosis, n (%)	73 (38.8%)	57 (58.2%)	16 (17.8%)	<0.01
CTP score	5.50 ± 1.09	5.54 ± 1.19	5.46 ± 0.98	0.59

Abbreviations: n, number; HBV, hepatitis B virus; HBeAg, hepatitis B e antigen; ALT, alanine aminotransferase; INR, international normalized ratio; CTP, Child–Turcotte–Pugh; Cr, creatinine; HVL, high viral load; PT, prothrombin time.

**Table 2:** Virologic responses during 120 months of therapy in the Non-HVL and HVL groups.

	**All**	**Non-HVL**	**HVL**	***p***
Virologic Response Rates *				
6 months	72/188 (38.3)	57/98 (58.2)	15/90 (16.7)	<0.01
12 months	120/182 (65.9)	82/94 (87.2)	38/88 (43.2)	<0.01
24 months	138/170 (81.2)	83/89 (93.3)	55/81 (67.9)	<0.01
36 months	137/154 (89.0)	78/81 (96.3)	59/73 (80.8)	<0.01
48 months	124/140 (89.0)	73/76 (96.1)	51/64 (79.7)	<0.01
60 months	117/128 (91.4)	67/69 (97.1)	50/59 (84.7)	0.01
120 months	99/109 (90.8)	57/59 (96.6)	42/50 (82.7)	0.02

* Data presented in parenthesis are the percentage of patients who showed virologic response out of the number of patients who were followed up at each time point. Abbreviations: HVL, high viral load.

**Table 3:** Virologic responses during 120 months of therapy in early VR and PVR groups at 6 months.

	All	Early VR6 ^§^ (n = 71)	PVR6 ^§^ (n = 111)	*p*
Virologic Response Rates *				
12 months	120/182 (65.9)	69/71 (97.2)	51/111 (45.9)	<0.01
24 months	138/170 (81.2)	65/68 (95.6)	73/102 (71.6)	<0.01
36 months	137/154 (89.0)	60/61 (98.4)	77/93 (82.8)	<0.01
48 months	124/140 (88.6)	55/57 (96.5)	69/83 (83.1)	0.01
60 months	117/128 (91.4)	55/55 (100)	62/73 (84.9)	<0.01
120 months	99/109 (90.8)	47/47 (100)	52/62 (83.9)	<0.01

* Data presented in parenthesis are the percentage of patients who showed virologic response out of the number of patients who were followed up at each time point. **^§^** Defined based on the viral response at the 6-month time point. Abbreviations: PVR, partial virologic response; VR, virologic response.

**Table 4:** Univariate and multivariate analysis of factors predictive of virologic response at 120 months.

	Univariate Analysis			Multivariable Model 1 *			Multivariable Model 2 *		
	OR	95% CI	*p*	aOR	95% CI	*p*	aOR	95% CI	*p*
Sex	0.41	0.08–2.04	0.28						
Age (years)	1.00	0.95–1.06	0.89						
HBV DNA > 7 (log_10_ IU/mL)	0.16	0.03–0.80	0.02	0.15	0.02–0.82	0.02			
ALT (IU/L)	1.00	0.99–1.00	0.70						
Total bilirubin	0.92	0.71–1.20	0.57						
HBeAg-positivity	0.28	0.05–1.37	0.11						
Albumin	5.62	1.65–19.08	<0.01	5.08	0.95–27.14	0.05	4.67	0.85–25.46	0.07
PT, INR	0.07	0.00–1.04	0.05	0.54	0.01–25.02	0.75	0.94	0.02–34.42	0.97
PVR at 6 months ^§^	1.00	NA	NA						
VR at 12 months	6.52	1.61–26.35	<0.01				5.65	1.33–23.97	0.01

* Multivariable model 1 adjusted for variables of status at treatment start; model 2 adjusted for other variables except presence of HVL at treatment start. ^§^ Cox analysis not feasible for the association between PVR at 6 months and VR at 120 months, as 100% of the early VR6 group demonstrated VR at 120 months. Abbreviations: HBeAg, hepatitis B e antigen; ALT, alanine aminotransferase; INR, international normalized ratio; PVR, partial virologic response; VR, virologic response; PT, prothrombin time; OR, odds ratio; aOR, adjusted odds ratio; CI, confidence interval; NA, not available

## Data Availability

The data that support the findings of this study are available from the corresponding author upon reasonable request. Access to data is restricted and will be considered on a case-by-case basis, subject to institutional review and applicable regulations.

## Supplementary Materials

The following supporting information can be downloaded at https://www.mdpi.com/article/10.3390/microorganisms13020218/s1: Figure S1: Patient flow diagram; Figure S2: Serologic response during 120 months in the non-HVL and HVL groups; Figure S3: Comparison of virologic response at 120 months based on the presence of HVL and PVR6; Table S1: Virologic Responses During 120 Months of Therapy in the Non-HVL and HVL Groups.
