# Supplementary material for: Need of Treatment Modification During Entecavir Therapy in Patients with Chronic Hepatitis B: Long-Term Follow-Up Results for 120 Months
Source: Microorganisms. 2025 Jan 21;13(2):218. doi: 10.3390/microorganisms13020218 (PMC11857378; doi:10.3390/microorganisms13020218)

Supplementary Material

Need of Treatment Modification During Entecavir Therapy in Patients with Chronic Hepatitis B: Long-Term Follow-Up Results for 120 Months

Contents

Table S1. Biochemical responses during 120 months of therapy in the non-HVL and HVL groups p. 2

Figure S1. Patient flow diagram p. 3

Figure S2. Serologic response during 120 months in the non-HVL and HVL groups. p. 4

Figure S3. Comparison of virologic response at 120 months based on the presence of HVL and PVR6 p. 6

Supplementary Table S1. Biochemical responses during 120 months of therapy in the non-HVL and HVL groups

	All	Non HVL	HVL	*P*-value
Biochemical Response rates*				
6 months	147/188 (78.2)	77/98 (78.6)	70/90 (77.8)	0.89
12 months	153/182 (84.1)	81/94 (86.2)	72/88 (81.8)	0.42
24 months	155/172 (90.1)	81/90 (90.0)	74/82 (90.2)	0.95
36 months	141/153 (92.2)	77/81 (95.1)	64/72 (88.9)	0.15
48 months	131/139 (94.2)	72/76 (94.7)	59/63 (93.7)	0.78
60 months	110/129(85.3)	61/71 (85.9)	49/58 (84.5)	0.81
120 months	95/104 (91.3)	55/62 (88.7)	40/42 (93.3)	0.24

* Data presented in parenthesis are the percentage of patients who showed biochemical response out of the number of patients who were followed up at each time point.

Abbreviations: HVL, high viral load.

Supplementary Figures

Supplementary Figure. S1. Patient flow diagram

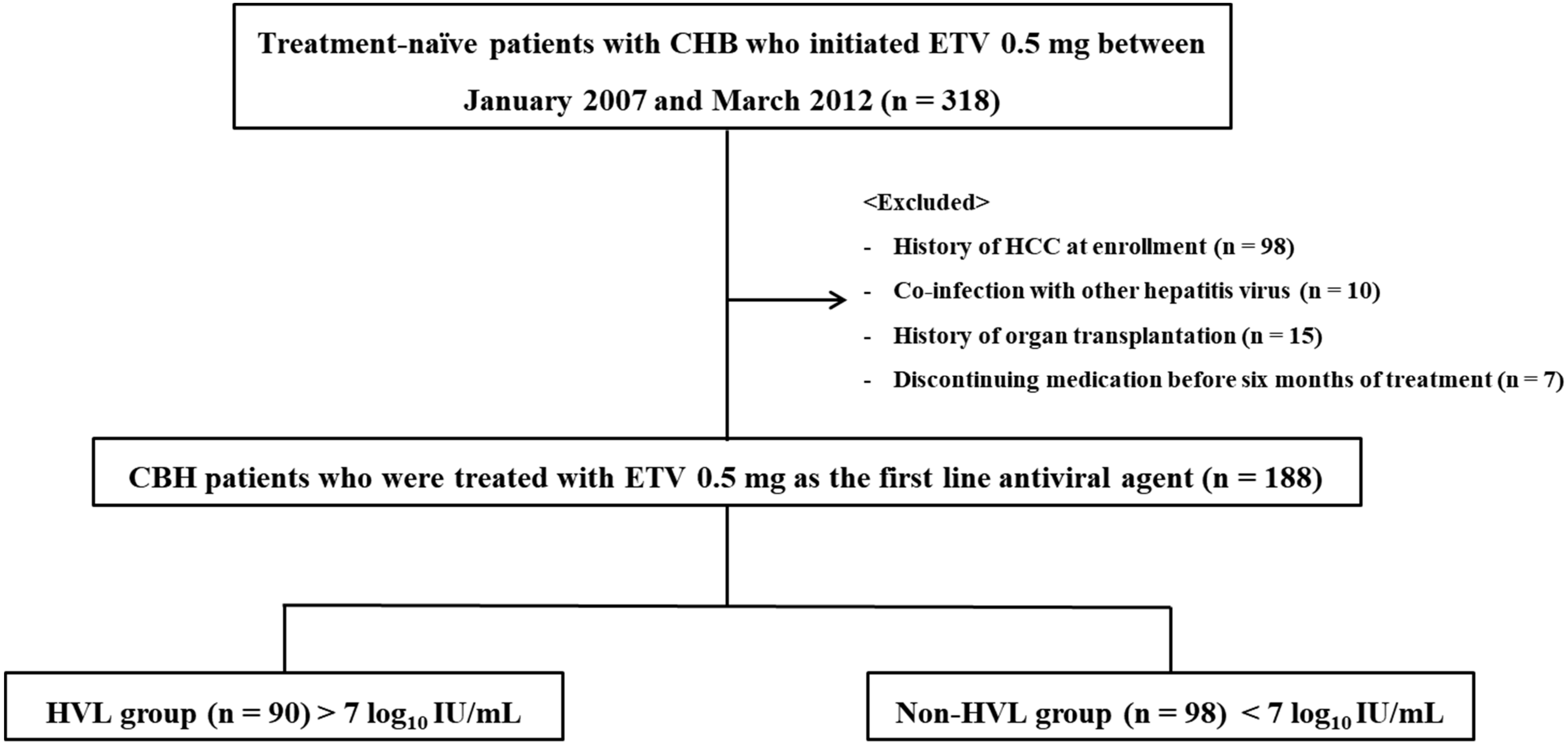

Supplementary Figure. S2. Serologic response during 120 months in the non-HVL and HVL groups. (A) Seroconversion (%) and (B) HBeAg loss (%) in HVL and non-HVL groups.

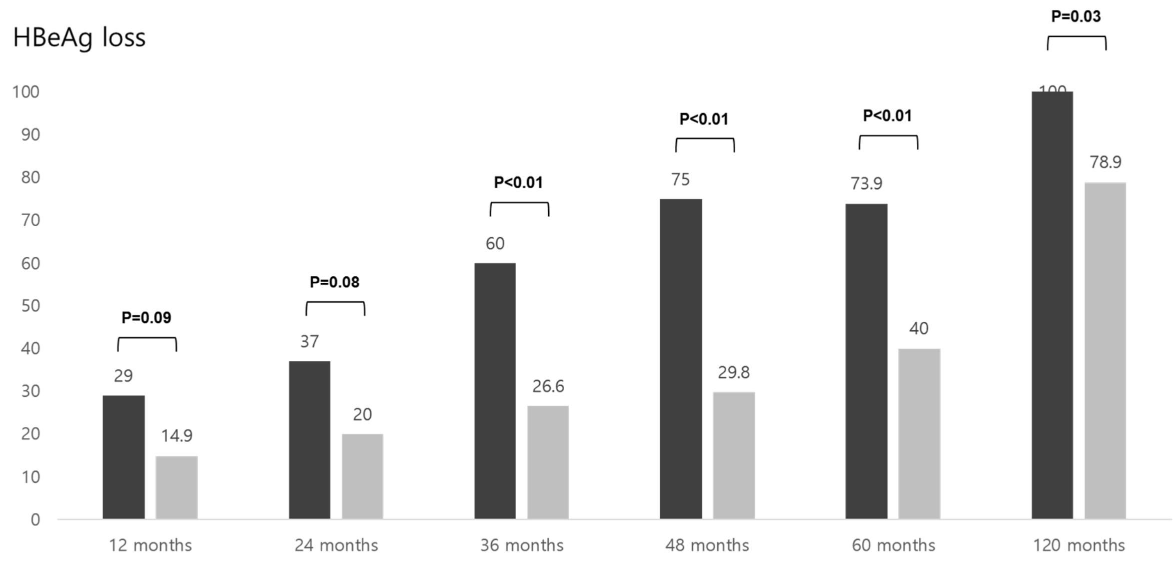

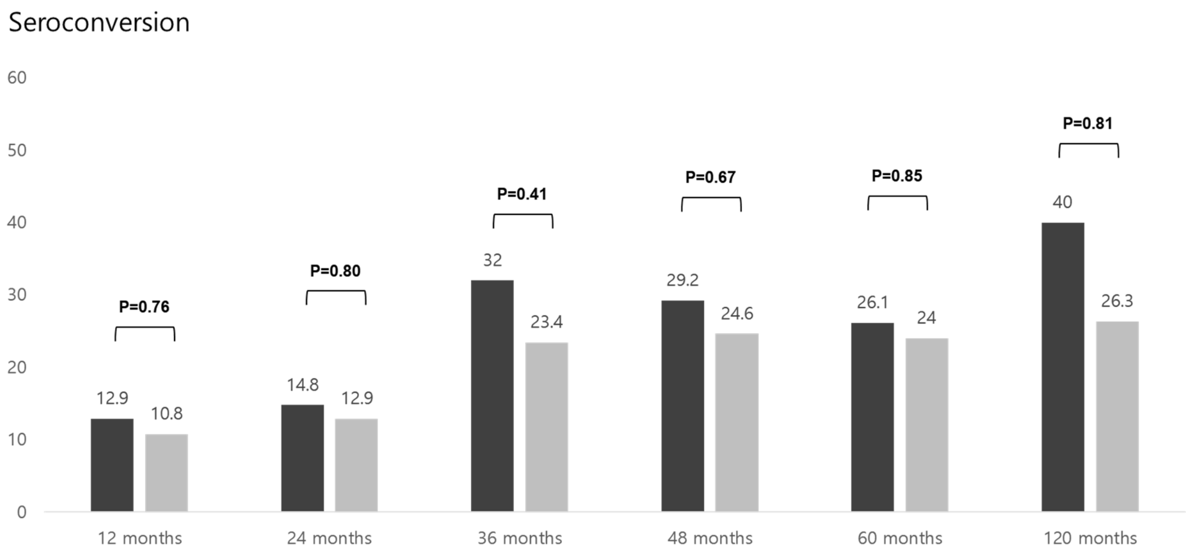
Seroconversion
60
50
40
30
20
10
0
P=0.76
P=0.80
P=0.41
P=0.67
P=0.85
P=0.81
12.9
10.8
14.8
12.9
32
23.4
29.2
24.6
26.1
24
40
26.3
12 months
24 months
36 months
48 months
60 months
120 months

Supplementary Figure. S3. Comparison of virologic response at 120 months based on the presence of HVL and PVR6

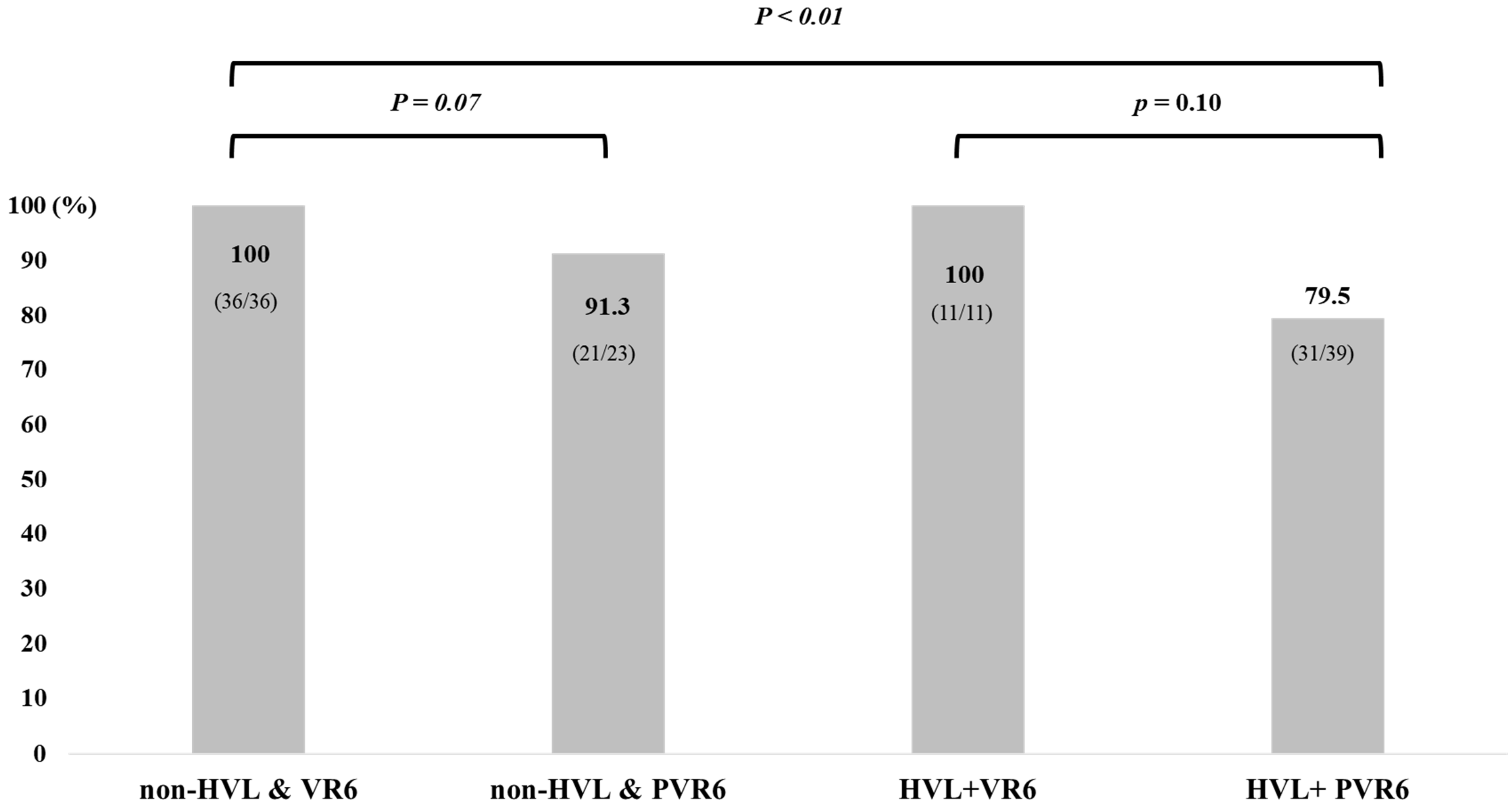

Supplement: Supplementary file 1 [file microorganisms-13-00218-s001.zip › microorganisms-3414908-supplementary.pdf]
